# The Management of a Case With Mucin-Producing Adenocarcinoma Originating From the Urachus

**DOI:** 10.7759/cureus.52376

**Published:** 2024-01-16

**Authors:** Panagiotis Angelopoulos, Sotirios Kapsalos- Dedes, Ioannis Manolitsis, Stamatios Katsimperis, Themistoklis Bellos, Ioannis Kyriazis, Panagiotis Neofytou, Lazaros Tzelves, Marinos Berdempes, Andreas Skolarikos

**Affiliations:** 1 Urology, Second Department of Urology, National and Kapodistrian University of Athens, Sismanogleio General Hospital, Athens, GRC; 2 Urology, Second Department of Urology, National and Kapodistrian University of Athens, Athens, GRC; 3 Urology, Second Department of Urology, National and Kapodistrian University of Athens, Sismanogleio General Hospital, Athens, Greece, Athens, GRC; 4 Urology, Second Department of Urology, National and Kapodistrian University of Athens, Sismanoglio General Hospital, Athens, GRC; 5 Urology, Second Department of Urology, National and Kapodistrian University of Athens, Sismanogleio General Hospital, Athens, GRC, Athens, GRC

**Keywords:** urology and oncology, adenocarcinoma, urachal cancer, pelvic lymph node dissection, cystectomy

## Abstract

Urachal cancer is a rare and aggressive type of cancer, frequently characterized by a lack of prominent symptoms. We herein report a case of a 50-year-old female with mucin-producing adenocarcinoma originating from the urachus who underwent partial cystectomy and the patient remains disease-free for 30 months after treatment.

## Introduction

Urachal cancer is a rare and aggressive form of cancer that arises in the urachus, a structure that connects the bladder to the umbilicus during fetal development. The urachus usually degenerates and becomes a fibrous band known as the median umbilical ligament after birth [1-3]. However, in some cases, cells in this area can undergo malignant transformation, leading to urachal cancer. One of the challenging aspects of urachal cancer is that it often presents with subtle or nonspecific symptoms in its early stages [3].

We hereby present a documented case wherein a patient underwent partial cystectomy to address the condition of urachal adenocarcinoma. Thirty months post-surgery, the patient remains free of the disease following the surgical procedure.

## Case presentation

A 50-year-old woman, a non-smoker with a medical history notable for diabetes mellitus, has been referred to the outpatient clinic due to the persistence of recurrent urinary tract infections over the course of the past year. Upon clinical evaluation, urine culture and ultrasound imaging were recommended. The urine culture results indicated the presence of Escherichia coli. Subsequently, the patient received treatment with a second-generation cephalosporin due to a documented quinolone allergy. Unfortunately, there was no observed improvement in symptoms, mirroring previous instances of treatment.

**Figure 1:**
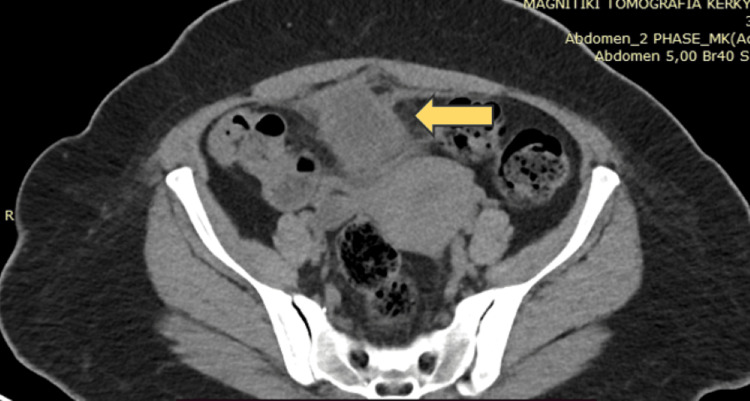
CT scan revealed a lesion suggestive of possibly cystic tissue

Ultrasound examination revealed the presence of a lesion within the urinary bladder. Consequently, the patient was advised to undergo a more comprehensive diagnostic assessment, specifically a contrast-enhanced computed tomography (CT) scan of the upper and lower abdomen (a CT urography protocol). The CT scan, with intravenous contrast agent administration, revealed a lesion suggestive of possibly cystic tissue (Figure 1). This lesion was located anteriorly and superiorly to the bladder, positioned between the bladder and the anterior abdominal wall. The proximity of the lesion to the bladder and the potential inflammatory origin of the lesion were noted. Further investigation and consultation are recommended to ascertain the nature and appropriate management of this identified pathology.

**Figure 2:**
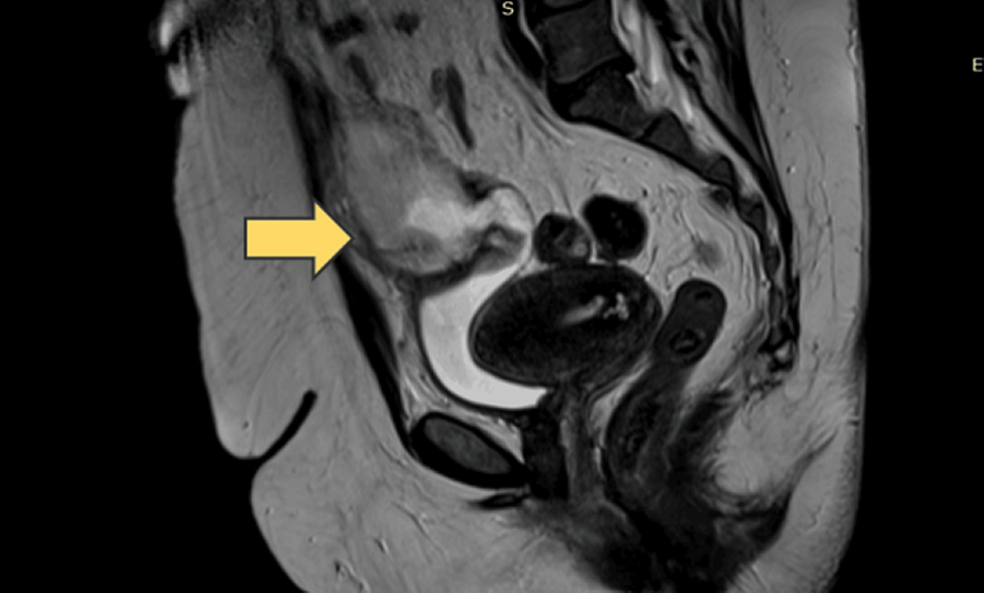
The MRI demonstrated a cystic lesion

Consequently, the patient was recommended to undergo a further MRI scan of the upper and lower abdomen, and arrangements were made for cystoscopy and colonoscopy. The subsequent colonoscopy, however, did not reveal any pathological changes in the large intestine. Thus, it became less likely that the morphoma was originating from or connected to the gastrointestinal tract. The MRI, conducted with the administration of a paramagnetic substance, demonstrated an enlargement of the cystic lesion at eight months from 4.8 cm × 3.6 cm to 8.6 cm × 7.3 cm (Figure 2). Notably, the lesion was observed to communicate with the lumen of the bladder and extend upwards to the level of the umbilicus. The walls of the lesion exhibited thickening, with localized enrichment of the parenchymal substance, and were further characterized by the presence of mural appendages and septum. The described cystic alteration is in contact with the anterior abdominal wall without evidence of permeation. The differential diagnosis suggests the possibility of a neoplastic lesion, with the inflammatory cyst of the urachus being less likely. Pathologically enlarged lymph nodes in the abdomen were not detected.

**Figure 3:**
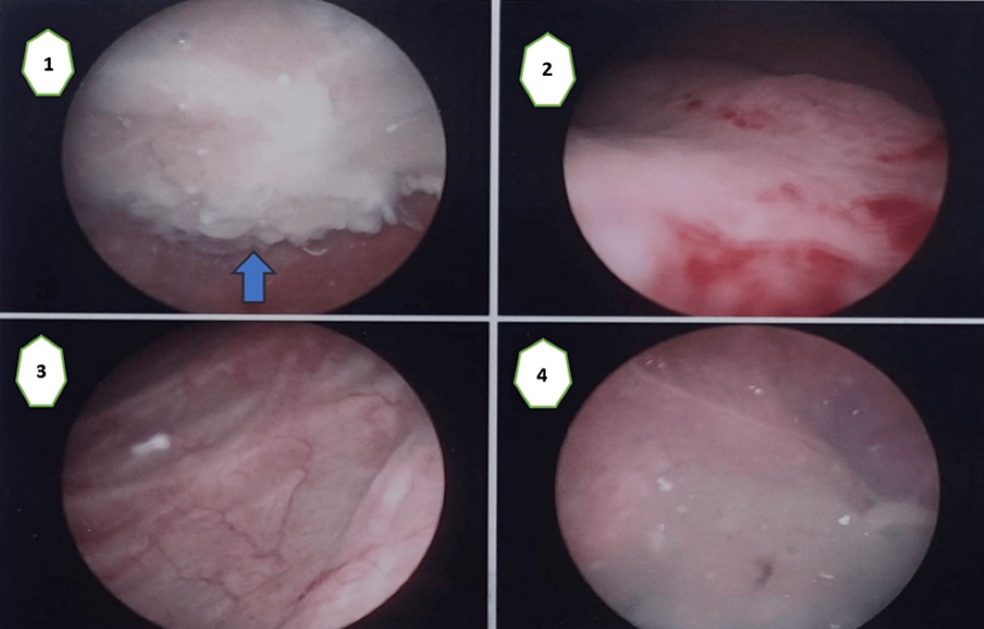
The images of the cystoscopy (1) A lesion within the urinary bladder; (2) the anatomic site of the bladder trigone; (3) left lateral wall of the bladder; (4) right lateral wall of the bladder.

During the cystoscopy, the existence of a modification inside the urinary bladder was verified. This alteration seems to extend from the posterior upper wall (Figure 3).

**Figure 4:**
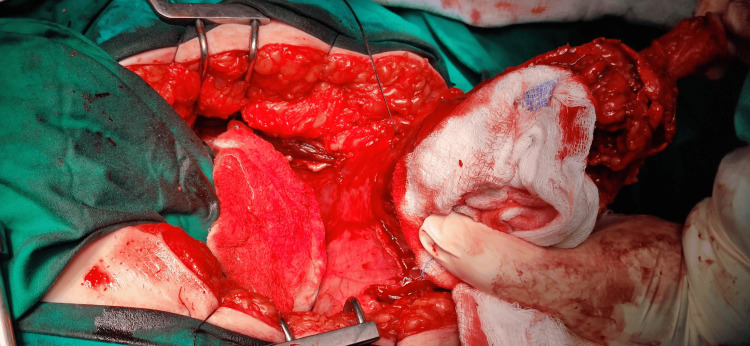
Image from the surgery

Furthermore, the patient underwent chest computed tomography, which revealed no pathological alterations. After completing the imaging assessment, the patient underwent surgical intervention and was admitted to the urology clinic. The patient underwent partial cystectomy with en bloc resection of the middle umbilical ligament and umbilicus and bilateral pelvic lymph node dissection (Figure 4). A surgical procedure involved making an incision located in the mid-supra-subumbilical region. Intraoperatively, the pathological alteration was identified, and a specimen was submitted for a quick biopsy, revealing the existence of adenocarcinoma. It was removed, along with a section of the urinary bladder around it.

**Figure 5:**
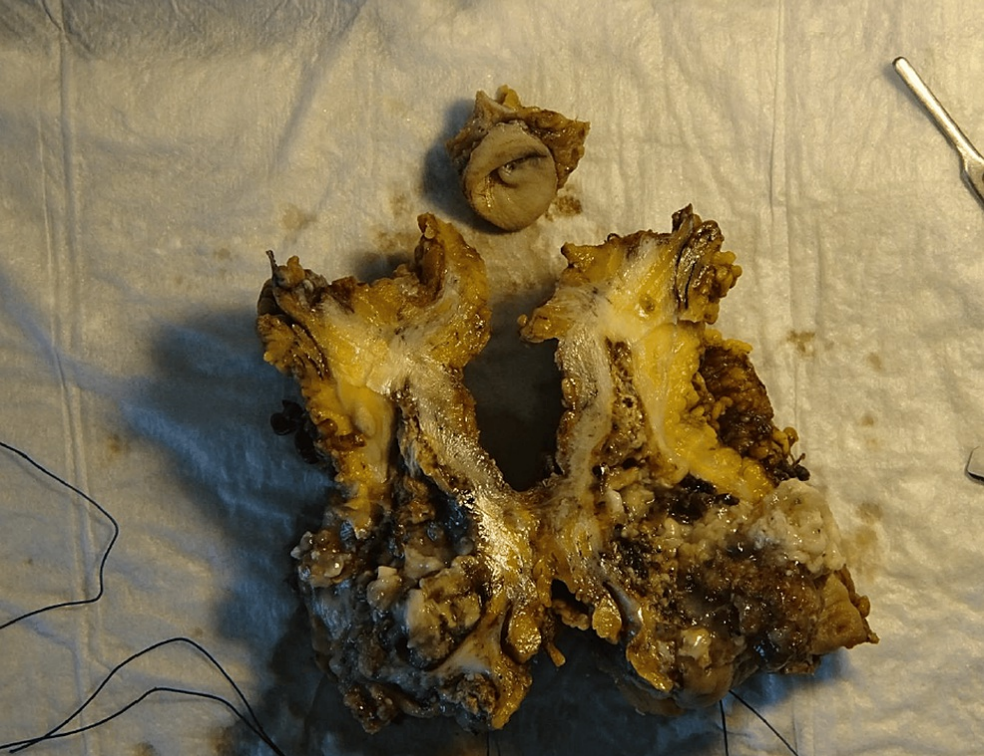
The formulation for histological examination

The patient experienced an uneventful postoperative course and was discharged on the fifth day after surgery. The histological examination revealed mucin-producing adenocarcinoma (Sheldon staging pT3) originating from the urachus (Figure 5), based on the localization and immunomorphological characteristics. Immunophenotyping revealed: CDX 2(+),CK20 (+), CK7 (+), p63(-), GATA3(-).

Adjuvant chemotherapy was recommended for the patient, but she declined the treatment. At the six-month mark, the patient underwent a cystoscopy, which did not reveal any recurrence of the disease. Now, 30 months post-surgery, the patient remains disease-free and is under surveillance with imaging checks every six months. She exhibits only frequent urination due to reduced bladder capacity, a symptom that does not affect her quality of life.

## Discussion

The urachus is the residual structure resulting from the regression of both the allantois and the ventral cloaca during embryonic development [1,2]. In the initial stages of embryonic development (between days 16 and 24), the allantois, originating from the vitelline sac, connects to the upper section of the ventral or anterior cloaca, creating the allantois channel. By the fifth week, the bladder takes shape from the middle part of the allantois, while the remaining portion, known as the cloaca, transforms into the urachus [2,3]. Although there was some disagreement regarding whether the urachus originates from the allantois or the cloaca, contemporary evidence indicates that it indeed arises from the cloaca [3].

Tumors affecting the urachus are frequently observed in males, whereas benign conditions like urachal cysts tend to be more prevalent in female patients [4]. Urachal cancer is an uncommon and aggressive form of cancer, often presenting with few noticeable symptoms, attributed to its location outside the bladder and peritoneal cavity [5]. It constitutes less than 0.5% of bladder cancers and a mere 0.01% of all adult malignancies. Typically affecting the bladder dome, urachal cancer is predominantly identified histologically as adenocarcinoma in 80-90% of cases, despite the fact that the usual lining of the urachus is transitional epithelium [3,6].

Adenocarcinomas constitute over 90% of urachal carcinomas, and the majority of these cases are invasive, exhibiting a striking similarity to adenocarcinomas from diverse sources. This resemblance complicates the diagnostic process. Despite the challenges of reaching a conclusive diagnosis, immunohistochemistry can be crucial in identifying urachal carcinoma. Typically, biomarkers such as CK20 and CDX2 are prominently expressed in most cases, while CK7 and β-catenin exhibit a lower frequency of expression [7].

Surgery serves as the primary treatment for localized and surgically manageable cases, though approximately one-third of patients are deemed unresectable upon initial presentation [6]. The recommended surgical approach often involves a partial cystectomy coupled with an en-bloc urachectomy and umbilectomy. This procedure entails the removal of the medial umbilical ligament from the bladder dome to the umbilicus, ensuring negative surgical margins [3,8]. If deemed necessary to achieve negative margins or if the extent of resection poses a risk of an insufficient functional urinary reservoir, it is advisable to contemplate a comprehensive cystectomy coupled with en-bloc resection of the urachal ligament [8]. In conclusion, there is no observable survival advantage linked to a complete cystectomy compared to a partial cystectomy [9].

The diagnosis is commonly established through cystoscopy and transurethral resection of the tumor, which confirms the presence of adenocarcinoma in the midline of the bladder. Radiographic assessments using CT or magnetic resonance imaging offer substantial supportive evidence [8]. A distinctive indication is the identification of a midline mass within the bladder, exhibiting either solid or cystic characteristics, particularly if accompanied by small calcifications, which is regarded as a pathognomonic finding.

Macroscopic or microscopic hematuria is a common clinical presentation observed in approximately 80% of patients, indicating that the tumor has breached the muscularis mucosae and invaded the urothelial surface [3,5]. Additional, albeit less frequent, symptoms at the time of presentation may include bacteriuria, mucinuria, pain, the presence of an abdominal mass, and infection around the umbilicus [3].

In contrast to conventional urothelial tumors, which originate from the surface epithelium and progress outward, urachal tumors emerge within the muscle wall or external to the bladder and progress inward. Consequently, the standard staging system employed for urothelial tumors is not applicable [10]. A comprehensive staging system, initially introduced by Sheldon (Table 1), was proposed to address urachal tumors. Unfortunately, a significant number of patients are diagnosed at stage III or beyond, likely because symptoms are often absent until tumors reach a considerable size or invade through the muscle wall and surface epithelium, prompting noticeable signs such as recurrent gross hematuria [10,11].

**Table 1:** The Sheldon staging system

Stage I	No invasion beyond the urachal mucosa
Stage II	Invasion confined to the urachus
Stage III(A)	Local extension into the bladder
Stage III(B)	Local extension into the abdominal wall
Stage III(C)	Local extension into the peritoneum
Stage III(D)	Extension to local viscera
Stage IV(A)	Metastases to regional lymph nodes
Stage IV(B)	Distant metastases

In 1984, Sheldon et al. [11] proposed a staging system (Table 1), which remains the most frequently referenced classification despite lacking formal validation. Molina et al. from the Mayo Clinic introduced another system (Table 2), offering an alternative perspective based on the analysis of 66 patients [3].

**Table 2:** Mayo Clinic staging system

Stage I	Confined to the urachus and/or the bladder
Stage II	Extension beyond the muscular layer of the urachus and/or the bladder
Stage III	Metastases to regional lymph nodes
Stage IV	Metastases to non-regional lymph nodes or other distant sites

The five prognostic factors that are commonly cited and widely validated include the stage of the disease, the presence of positive margins post-surgery, the pathological tumor grade, the presence of positive lymph nodes, and the type of surgery [12,13].

Utilizing the TNM staging system as outlined earlier, Molina et al. from Mayo Clinic demonstrated that the median survival exceeds 10 years for stage I and is approximately 7.5 years for stage II. However, there is a notable decline in survival, dropping to one to two years for stage III and less than one year for stage IV, indicating a significant impact of disease progression on the overall prognosis [3].

## Conclusions

In summary, urachal carcinoma represents a rare and challenging cancer with a generally poor prognosis. Surgical intervention remains the cornerstone of treatment, underscoring the significance of achieving a comprehensive urachectomy, inclusive of umbilectomy, and securing negative surgical margins to enhance long-term survival. Pathologic factors play a pivotal role in predicting patient outcomes.

## References

[1] Beck AD, Gaudin HJ, Bonham DG (1970). Carcinoma of the urachus. Br J Urol.

[2] Cappele O, Sibert L, Descargues J, Delmas V, Grise P (2001). A study of the anatomic features of the duct of the urachus. Surg Radiol Anat.

[3] Molina JR, Quevedo JF, Furth AF, Richardson RL, Zincke H, Burch PA (2007). Predictors of survival from urachal cancer: a Mayo Clinic study of 49 cases. Cancer.

[4] Mekras GD, Block NL, Carrion HM, Ishikoff M (1980). Urachal carcinoma: diagnosis by computerized axial tomography. J Urol.

[5] Hamilou Z, North S, Canil C (2020). Management of urachal cancer: a consensus statement by the Canadian Urological Association and Genitourinary Medical Oncologists of Canada. Can Urol Assoc J.

[6] Van Allen J (2021). A rare case of urachal adenocarcinoma with bone marrow metastasis. BMJ Case Rep.

[7] Kumar R, Harilal S, Abdelgawad MA, Ghoneim MM, Kumar A, Mathew B (2023). Urachal carcinoma: the journey so far and the road ahead. Pathol Res Pract.

[8] Siefker-Radtke A (2012). Urachal adenocarcinoma: a clinician's guide for treatment. Semin Oncol.

[9] Henly DR, Farrow GM, Zincke H (1993). Urachal cancer: role of conservative surgery. Urology.

[10] Siefker-Radtke A (2006). Urachal carcinoma: surgical and chemotherapeutic options. Expert Rev Anticancer Ther.

[11] Sheldon CA, Clayman RV, Gonzalez R, Williams RD, Fraley EE (1984). Malignant urachal lesions. J Urol.

[12] Wright JL, Porter MP, Li CI, Lange PH, Lin DW (2006). Differences in survival among patients with urachal and nonurachal adenocarcinomas of the bladder. Cancer.

[13] Bruins HM, Visser O, Ploeg M, Hulsbergen-van de Kaa CA, Kiemeney LA, Witjes JA (2012). The clinical epidemiology of urachal carcinoma: results of a large, population based study. J Urol.

